# Effects of long-term and brain-wide colonization of peripheral bone marrow-derived myeloid cells in the CNS

**DOI:** 10.1186/s12974-020-01931-0

**Published:** 2020-09-20

**Authors:** Lindsay A. Hohsfield, Allison R. Najafi, Yasamine Ghorbanian, Neelakshi Soni, Edna E. Hingco, Sung Jin Kim, Ayer Darling Jue, Vivek Swarup, Mathew A. Inlay, Kim N. Green

**Affiliations:** 1 Department of Neurobiology and Behavior, University of California, 3208 Biological Sciences III, Irvine, CA 92697-4545 USA; 2 Sue and Bill Gross Stem Cell Research Center, University of California, Irvine, CA 92697 USA; 3 Department of Molecular Biology and Biochemistry, University of California, Irvine, CA 92697 USA

**Keywords:** Microglia, Monocytes, CSF1R inhibition, Irradiation, Bone marrow transplant, Brain

## Abstract

**Background:**

Microglia, the primary resident myeloid cells of the brain, play critical roles in immune defense by maintaining tissue homeostasis and responding to injury or disease. However, microglial activation and dysfunction has been implicated in a number of central nervous system (CNS) disorders, thus developing tools to manipulate and replace these myeloid cells in the CNS is of therapeutic interest.

**Methods:**

Using whole body irradiation, bone marrow transplant, and colony-stimulating factor 1 receptor inhibition, we achieve long-term and brain-wide (~ 80%) engraftment and colonization of peripheral bone marrow-derived myeloid cells (i.e., monocytes) in the brain parenchyma and evaluated the long-term effects of their colonization in the CNS.

**Results:**

Here, we identify a monocyte signature that includes an upregulation in *Ccr1*, *Ms4a6b*, *Ms4a6c*, *Ms4a7*, *Apobec1*, *Lyz2*, *Mrc1*, *Tmem221*, *Tlr8*, *Lilrb4a*, *Msr1*, *Nnt*, and *Wdfy1* and a downregulation of *Siglech*, *Slc2a5*, and *Ccl21a/b*. We demonstrate that irradiation and long-term (~ 6 months) engraftment of the CNS by monocytes induces brain region-dependent alterations in transcription profiles, astrocytes, neuronal structures, including synaptic components, and cognition. Although our results show that microglial replacement with peripherally derived myeloid cells is feasible and that irradiation-induced changes can be reversed by the replacement of microglia with monocytes in the hippocampus, we also observe that brain-wide engraftment of peripheral myeloid cells (relying on irradiation) can result in cognitive and synaptic deficits.

**Conclusions:**

These findings provide insight into better understanding the role and complexity of myeloid cells in the brain, including their regulation of other CNS cells and functional outcomes.

## Background

Microglia are the primary resident immune cells of the brain and possess the ability to rapidly proliferate and migrate in response to damage, injury, or infection. These immune detection and defense functions place microglia at the frontline of repair and recovery in the central nervous system (CNS). Microglia constitute 5–10% of all brain cells and are spatially arranged in a tile-like manner throughout the parenchyma—each cell inhabits a discrete territory in which its processes constantly retract and extend to survey the local environment [1], detecting and responding to stimuli accordingly. Microglia derive from yolk sac primitive macrophages, which enter and colonize the brain as immature microglia during embryonic development (~ E9.5) [2, 3]. At E13.5, formation of the blood-brain barrier (BBB) effectively isolates the brain, and thus from this point on and throughout adulthood, microglial population maintenance relies on local self-proliferation (under steady state conditions), independent of contributions from bone marrow (BM)-derived myeloid cells. Under inflammatory or disease conditions, studies have shown that BM-derived peripheral myeloid cells (i.e., monocytes) enter the brain, but do not differentiate into microglia or contribute significantly to the resident CNS microglial pool unless under exceptional circumstances (i.e., BM transplantation) [2, 4, 5]. However, even under these circumstances (i.e. BM transplant models), only a fraction of microglia derive from BM sources [6].

Microglia are dependent on signaling through the colony-stimulating factor 1 receptor (CSF1R) for their survival across the lifespan. We previously identified several selective CSF1R inhibitors that are orally bioavailable and CNS-penetrant and demonstrated that administration of these inhibitors results in rapid elimination of the murine microglial compartment [7–10]. Investigations on the effects of CSF1R inhibitors in primates [11] and humans [12, 13] are ongoing, but provide a promising therapeutic strategy for brain disorders affected by microglial dysfunction [14]. One of the inhibitors that we first identified, pexidartinib (PLX3397), recently received FDA approval for the treatment of tenosynovial giant cell tumor [15].

Due to their central role in the CNS and several brain disorders, microglia have become attractive therapeutic targets; thus, strategies that replace or manipulate these cells could provide broad-reaching implications. In addition to depletion, we previously demonstrated that following microglial elimination, withdrawal of CSF1R inhibitors stimulates the rapid repopulation of the entire CNS with new microglia, and that these new cells derive from surviving cells within the CNS [7, 16–21]. This remarkable ability to deplete and then rebuild a cellular compartment throughout the brain parenchyma could provide a unique tool for developing cell and gene-based delivery systems to the brain. However, in previously utilized chimeric paradigms, we have found that transplanted exogenous myeloid cells are rapidly outcompeted by the surviving endogenous microglia. Engraftment of myeloid cells is severely limited in the brain unless performed under conditions inducing a depleted microglial niche (i.e., genetic loss of CSF1R), limiting the effectiveness and scope of transplanted engineered cells [22–25].

BM transplantations are often clinically utilized to replace the BM compartment with healthy donor BM cells. This technique typically relies on whole body irradiation to achieve myeloablation; however, recent studies have also indicated that myelosuppressive agents, such as the chemotherapeutic busulfan, offer viable alternatives [26]. We and others recently learned that combining CSF1R-dependent microglial elimination with whole body irradiation stimulates the complete repopulation of the CNS myeloid niche with BM-derived cells [22]. This technique not only provides us with a novel and clinically relevant tool to fully replace the microglial tissue with exogenous myeloid cells, but also allows for the potential unprecedented delivery of engineered myeloid cells to the brain (e.g., to carry a corrected mutation or therapeutic payload). Here, we sought to evaluate and characterize this method of complete microglial cell replacement utilizing CSF1R inhibition and BM transplantation and the long-term effects peripheral myeloid cells confer in the brain.

The role of peripheral-derived monocytes in disease-associated pathologies and neurological disorders remains under debate [27, 28]. Several studies have shown that monocyte infiltration into the brain is associated with injury, disease, and neuropsychiatric conditions, suggesting that monocytes contribute to neuronal injury and demyelination [29–34]. However, other studies have shown that peripheral myeloid cell engraftment after BM transplant is beneficial and in some diseases macrophages provide neuroprotection and promote recovery [35–40].

To understand how peripherally derived CNS-resident myeloid cells differ from resident microglia in terms of their effects on the brain, and as a proof-of-principle for a clinically feasible cell delivery system, we utilized a unique experimental paradigm combining whole body irradiation, BM transplant, and a CSF1R inhibitor (CSF1Ri) to investigate (1) whether full replacement of the microglial tissue can be achieved with BM-derived peripheral myeloid cells and (2) what are the long-term (~ 10 months after transplant; ~ 6 months after CSF1Ri treatment) consequences of this replacement on other CNS cell properties and structures, gene expression, and behavior and cognition.

## Methods

### Compounds

Pexidartinib (PLX3397) was provided by Plexxikon Inc. and formulated in AIN-76A standard chow at a dose of 600 ppm by Research Diets Inc.

### Mice

All mice were obtained from The Jackson Laboratory. For transplant studies, bone marrow cells were isolated from CAG-EGFP mice (006567). All other mice were male C57BL/6 (000664) mice. Animals were housed with open access to food and water under 12 h/12 h light-dark cycles. All mice were aged to 1.5 months unless otherwise indicated.

### Animal treatments

All rodent experiments were performed in accordance with animal protocols approved by the Institutional Animal Care and Use Committee at the University of California, Irvine (UCI). *Microglial depletion*: Mice were administered ad libitum with PLX3397 at a dosage of 600 ppm (to eliminate microglia) or vehicle (control) for 14 days. *Bone marrow transplant*: C57BL/6 mice were anesthetized with isoflurane and then irradiated with 1000 cGy (whole-body irradiated) and reconstituted via retroorbital injection with 2 × 10^6^ whole BM cells from CAG-EGFP mice*.* Blood was measured at ~ 12 weeks post-transplantation to track granulocyte chimerism. At the time of sacrifice, the mice were euthanized and BM was harvested and analyzed by flow cytometry for HSC chimerism. This established an average percent chimerism of 97% in all whole-body irradiated mice. *Tissue collection*: Following any treatments, mice were sacrificed via carbon dioxide inhalation and perfused transcardially with 1X PBS. The brains were extracted and dissected down the midline, with one half flash-frozen for subsequent RNA and protein analyses and the other half drop-fixed in 4% paraformaldehyde. Fixed brains were cryopreserved in PBS + 0.05% sodium azide + 30% sucrose, frozen, and sectioned at 40 μm on a Leica SM2000 R sliding microtome for subsequent immunohistochemical analyses.

### Flow cytometry

Bone marrow/hematopoietic stem cells were extracted from femurs and tibia by flushing with PBS + 2% FBS. Peripheral blood cells/granulocytes were collected via the tail vein in EDTA. Red blood cells were lysed using 1x ACK Lysis Buffer. Cells were then stained for analysis by flow cytometry with the following surface antibodies purchased from Biolegend at 1:200 unless otherwise indicated: CD34-eFlour660 (1:50, 50-0341-80, eBioscience), Sca-1-AF700 (1:100, 108141), Ter119-PE/Cy5 (116209), ckit/CD117-PE/Cy7 (25-1171-81, eBioscience), CD150/SLAM-PerCP-eFlour710 (46-1502-82, eBioscience), CD11b-APC (101212), Gr1-AF700 (108422), CD45-APC/Cy7 (103116), NK1.1-PE (108707), and CD27-APC/Cy7 (124226). Flow cytometry analysis was performed using the BD LSRII.

### Behavioral and cognitive testing

Mouse behavior, motor function, and cognition was evaluated using the following tasks: elevated plus maze, open field, novel place recognition test, rotarod, sociability test, spontaneous alternation Y-maze, and contextual fear conditioning in the order listed, and as previously described unless otherwise indicated [7, 8, 16]. Testing was conducted at 1 and 6 months recovery (i.e., after CSF1R inhibitor removal and myeloid cell repopulation). *Sociability test*: Crawley’s or Three-Chamber Sociability test assesses general sociability, or time spent with another rodent. In brief, animals were placed in a Three-Chamber Sociability Test box (19 cm × 45 cm) with two dividing walls made of clear Plexiglas allowing free access to each chamber. During habituation, the subject mouse is placed in the middle chamber for 5 min for adaption. During testing (24 h after habituation), a stranger mouse (inside a wire containment cup) is placed in one of the side chambers, and the subject mouse is placed in the center chamber and allowed to access and explore all three chambers for 10 min. The placement of the stranger mouse in the left and right chambers is systemically altered between trials. The duration of time spent in each chamber, velocity, and distance traveled was measured. *Spontaneous alternation Y-maze*: For this task, mice were placed in a Y-maze (35.2 cm arm length × 5 cm width × 20 cm sidewall height). Each animal was allowed to freely explore the arena for 8 min. Distinct intra-maze visual cues were positioned at the end of each arm for spatial orientation. Spontaneous alternation, which measures the willingness of an animal to explore new environments, was measured by the number of triads, or entry of all three arms in a consecutive sequence (i.e., ABC and not BAB). *Contextual fear conditioning (CFC):* For the training trial, mice were placed in a CFC chamber (Ugo Basile; 17 cm length × 17 cm width × 25 cm height) and allowed to explore for 2 min. At 2 min, the animal received one shock (3 s, 0.5 mA). Following the cessation of the shock, the animal remained in the chamber for an additional 30 s before being returned to their home cage. The testing trial was conducted 24 h later, in which the animal was placed in the chamber and allowed to explore for 5 min. EthoVision Activity Analysis software was used to detect activity levels and freezing behaviors. Contextual memory was assessed by measuring freezing behaviors, defined as the total lack of body movement except for respiration. Unless otherwise indicated, behavioral readouts for all tasks were calculated from video using the EthoVision XT 14 tracking system (Noldus).

### Histology and confocal microscopy

Fluorescent immunolabeling followed a standard indirect technique as described previously [7, 41]. Free-floating brain sections were washed with 1x PBS, incubated in normal serum blocking solution (5% normal serum + 0.2% Triton X-100 in 1x PBS) for 1 hr and then stained against primary antibodies overnight (~ 16–24 h) at 4 °C in normal serum blocking solution utilizing the following primary antibodies and dilutions: ionized calcium binding adaptor molecule 1 (IBA1; 1:1000; 019-19741, Wako and ab5076, Abcam), P2RY12 (1:200; HPA014518, Sigma-Aldrich), TMEM119 (1:200; ab209064, Novus), CD68 (1:1000; MCA1957GA, Bio-Rad), AXL (1:100, AF854, R&D Systems), Ki67 (1:200, ab16667, Abcam), glial fibrillary acidic protein (GFAP; 1:3000, ab4674, Abcam), S100β (1:200, ab52642, Abcam), microtubule-associated protein 2 (MAP 2; 1:500, ab32454, Abcam), NeuN (1:1000; MAB377, EMD Millipore), PSD95 (1:500, ab18258, Abcam), synaptic vesicle glycoprotein 2A (SV2A; 1:200; 119 002, Synaptic Systems), doublecortin (DCX; 1:500; sc-8066, Santa Cruz Biotechnology), IgG (1:200, 12-371, Millipore), and fibrinogen (1:1000, A0080, Dako). Following this, sections were washed with 1x PBS, incubated in fluorescent dye (Alexa Fluor)-conjugated secondary antibodies (1:200 in normal serum blocking solution) for 1 h and then washed in 1x PBS and mounted on slides. Prussian blue staining was performed using an Iron Stain Kit (ab150674, Abcam) according to manufacturer’s instructions with Nuclear Fast Red solution (N2030, Sigma-Aldrich). A spleen was used as a positive control illustrating how the protein-bound ferric iron pigment stains bright blue. High-resolution fluorescent images were obtained using a Leica TCS SPE-II confocal microscope and LAS-X software. Composite images were obtained by setting up z-stacks (× 20 objective: 5 μm thickness with ~ 8 sections; × 63 objective: 2 μm thickness with ~ 20 sections) and then ultimately collapsed into an overlay image. Post image processing was done using LAS-X software. All adjustments were kept consistent between groups. For confocal imaging, one field of view (FOV) per brain region was captured per mouse unless otherwise indicated. For whole brain stitches, automated slide scanning was performed using a Zeiss AxioScan.Z1 equipped with a Colibri camera and Zen AxioScan 2.3 software. Microglial morphology was determined using the filament module in Bitplane Imaris 7.5, as described previously [16]. Cell quantities were determined using the spots module in Imaris. Percent coverage measurements were determined in ImageJ (NIH).

### RNA sequencing and analysis

Total RNAs were extracted by using RNeasy Mini Kit (Qiagen). RNA integrity number (RIN) was measured, and samples with RIN ≥ 7.0 were kept for library construction. cDNA synthesis, amplification, library construction, and sequencing were performed by Novogene Inc. using Illumina NovaSeq and HiSeq platforms with paired-end 150 bp (PE 150) sequencing strategy. *Read alignment and expression quantification*: Pair-end RNA-seq reads were aligned using STAR v.2.5.1b with the options (--outFilterMismatchNmax 10 --outFilterMismatchNoverReadLmax 1 --outFilterMultimapNmax 10) [42]. Rsubread was used to generate feature counts [43]. Gene expression was measured using Limma, edgeR, and org.Mm.eg.db packages with expression values normalized as RPKM [44–47]. *Differential expression analysis*: Libraries with uniquely mapping percentages higher than 80% were considered to be of good quality and kept for downstream analysis. Protein coding and long non-coding RNA genes, with expression RPKM ≥ 1 in at least three samples, were collected for subsequent analysis. Differential expression analysis was performed by using Limma, edgeR, and org.Mm.eg.db [44–47]. Differentially expressed genes (DEGs) were selected by using false discovery rate (FDR) < 0.05. Top significant genes are displayed as a volcano plot constructed using GLimma, ggplot2, and EnhancedVolcano (FDR < 0.05, LogFC > 1) [48]. Normalized (min max normalization for each individual gene) log2-transformed expression values are displayed as a heatmap with hierarchical clustering utilizing pheatmaps. *Weighted correlation network analysis*: Network analysis was performed using weighted gene co-expression analysis (WGCNA) package in R [49]. First, bi-weighted mid-correlations were calculated for all gene pairs and then used to generate an eigengene network matrix, which reflects the similarity between genes according to their expression profiles. This matrix was then raised to power β (β = 18). Modules were defined using specific module cutting parameters (minimum module size = 100 genes, deepSplit = 4, and threshold of correlation = 0.2). Modules with a correlation greater than 0.8 were merged. We used first principal component of the module, called signed bicor network, to correlate brain region, irradiation, and treatment. Hub genes were defined using intra-modular connectivity (kME) parameter of the WGCNA package. *Gene enrichment analysis*: Gene set enrichment analysis was done using enrichR package 71. *Gene ontology and pathway analysis*: Specific modules were analyzed for Gene ontology (GO) enrichment by enrichR with corrected *p* values < 0.05. STRING interaction maps or functional protein association networks were generated using string-db.org (STRING Consortium 2020).

### Data analysis and statistics

Statistical analysis was performed with Prism Graph Pad (v.8.0.1). To compare two groups, the unpaired Student’s *t* test was used. To compare multiple groups, a one-way ANOVA with Tukey’s post hoc test was performed. For all analyses, statistical significance was accepted at *p* < 0.05. All bar graphs are represented as means ± SEM and significance expressed as follows: *p < 0.05, **p < 0.01, ***p < 0.001. *n* is given as the number of mice within each group.

## Results

### Combining irradiation, bone marrow transplant, and microglial depletion achieves long-term and complete peripheral myeloid cell engraftment in the brain

**Fig. 1:**
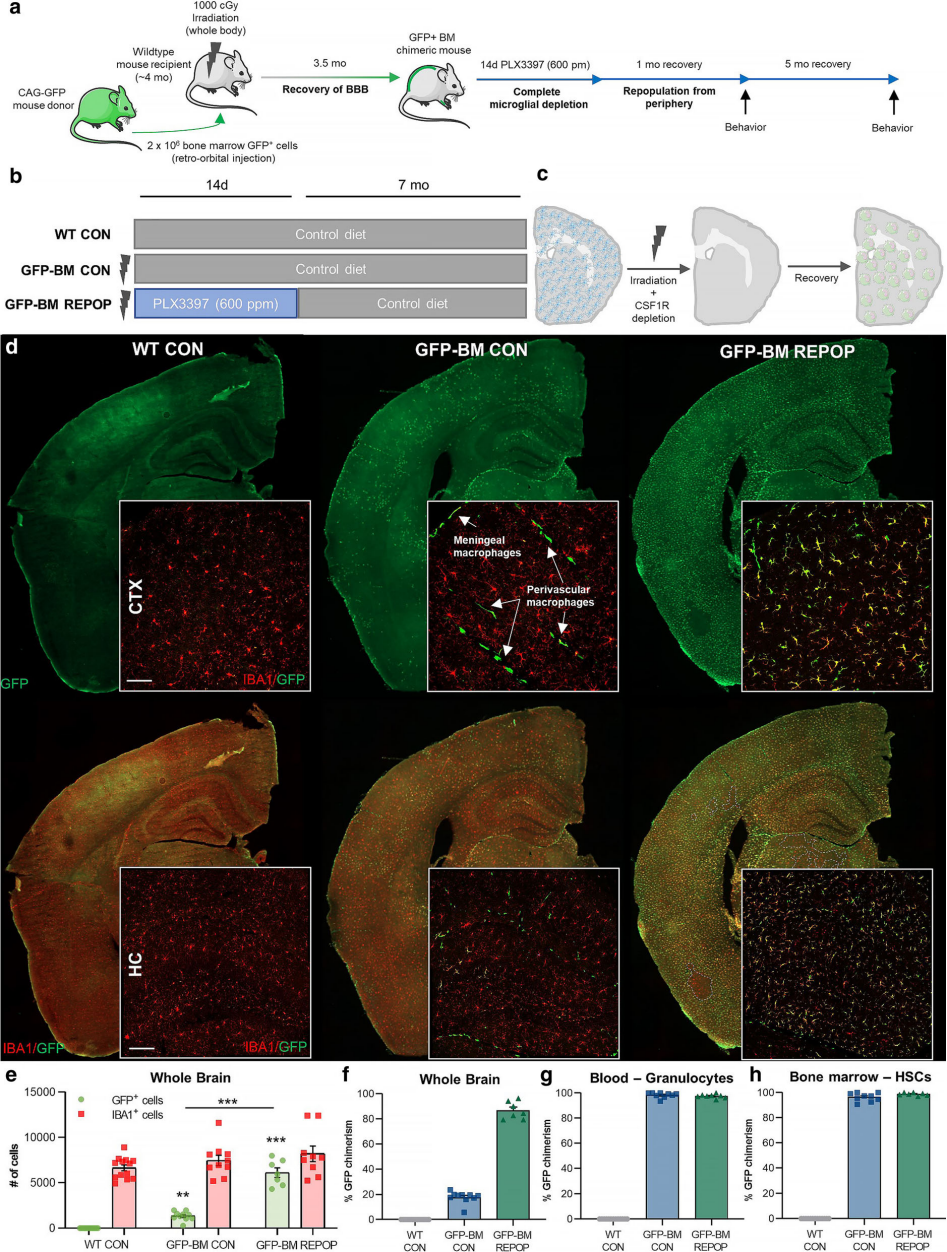
Combining irradiation, bone marrow transplant, and microglial depletion, achieves long-term and complete peripheral myeloid cell engraftment in the brain. **a**–**c** Experimental paradigm: Schematic depicting generation of chimeras with complete and long-term parenchymal engraftment of GFP^+^ bone marrow (BM)-derived peripheral myeloid cells in the brain, achieved by whole-body irradiation, BM transplant, and colony-stimulating factor 1 receptor (CSF1R)-dependent depletion. Four-month-old wild-type (WT) mice underwent whole-body irradiation (1000 cGy) and then BM transplant via retroorbital injection of 2 × 10^6^ BM cells from age- and sex-matched CAG-GFP mice. 3.5 months following BM transplant, mice were treated with 600 ppm of PLX3397 for 14 days, achieving microglial depletion, and then were placed on control diet for 6 months. **d** Representative whole brain images of GFP^+^ (green) and IBA1^+^ (red) cell deposition in control (WT CON), irradiated (GFP-BM CON), and monocyte-engrafted (GFP-BM REPOP) mice. Inserts include higher-resolution images of GFP^+^ engrafting cells, which appear exclusively in blood vessels and meninges in GFP-BM CON mice. **e** Quantification of number of GFP^+^ and IBA1^+^ cells in the whole brain. **f**–**h** Quantification of percent GFP chimerism in the whole brain (**f**), blood via granulocytes (CD45^+^NK1.1^−^CD11b^+^GR1^+^ cells) measured at ~ 12 weeks post irradiation/BM transplant (**g**), and bone marrow via hematopoietic stem cells (HSC; Ter119^−^CD27^+^ckit^+^Sca^+^CD150^+^CD34^−^ cells) measured at time of sacrifice (~ 10 months post irradiation/BM transplant; H). Data are represented as mean ± SEM (*n* = 8–9). ***p < 0.001. **p < 0.01. CTX cortex, HC hippocampus, GFP green fluorescent protein, IBA1 ionized calcium adapter molecule 1. Scale bar ~ 50 μm (top panel); ~ 100 μm (lower panel)

Here, we set out to explore as a proof-of-principle the clinical feasibility of a pharmacological-based method for near complete myeloid cell replacement. It is well known that BM-derived cells enter the brain following whole-body irradiation [50]; however, cell engraftment is limited. To this end, we and others [22] recently developed a unique paradigm, which utilizes whole-body irradiation, BM transplant, and a CSF1Ri treatment to achieve near complete parenchymal engraftment by donor peripheral myeloid cells (Fig. 1A–C). Previous studies have employed lead shielding of the head (i.e., body-only irradiation) to prevent peripheral-derived myeloid cell infiltration [22] and limit the effects of irradiation on the brain [51] caused during whole-body irradiation (i.e., head + body irradiation); however, we have found that head irradiation is required for optimal cell replacement. Although this method presents therapeutic potential for the replacement of dysfunctional myeloid cells, no studies to date have investigated the long-term consequences of such replacement. Given that microglia, non-parenchymal macrophages, and monocytes represent distinct heterogeneous populations of myeloid cells and it remains unclear whether these cells share overlapping functional capabilities, we set out to investigate the long-term consequences of microglial replacement with bone marrow (BM)-derived peripheral myeloid cells. To accomplish this, three experimental groups were generated: (1) WT CON, C57BL/6 wildtype (WT) control mice; (2) GFP-BM CON, WT mice subjected to whole body irradiation and retro-orbital infusion of donor CAG-green fluorescent protein (GFP) BM cells; and (3) GFP-BM REPOP, WT mice subjected to whole-body irradiation, GFP-BM transplant, and CSF1Ri-induced repopulation. It should be noted that WT CON received no irradiation, transplantation of GFP BM cells, or CSF1Ri treatment. Peripheral percent chimerism was evaluated after ~ 12 weeks via blood granulocytes and averaged ~ 98.0% in both irradiated groups (Fig. 1G). Previous studies have shown that high-dose cranial irradiation increases BBB permeability, albeit transiently [52], and so animals were provided with a 3.5 months BBB recovery period prior to further treatment. After 3.5 months, mice were treated for 14 days with the CSF1Ri PLX3397 at a dose of 600 ppm to deplete the microglial compartment. PLX3397 was then withdrawn, effectively stimulating myeloid cell repopulation. Under normal circumstances, repopulation of the microglial niche derives from the proliferation of surviving cells [7, 21, 53, 54], replacing the depleted tissue. However, in this study, we found that repopulation under exceptional conditions (i.e., following whole-body irradiation and microglial depletion) derives from BM-derived myeloid cells and results in the near complete replacement of the microglial niche with BM-derived peripheral myeloid cells (i.e., monocytes) within 14 days. These findings are in line with a previous study by Bruttger et al. showing that BM-derived macrophages repopulate the irradiated brain following genetic microglial depletion [54]. In their approach, Bruttger et al. utilize a genetic and toxin-based mouse model of microglial depletion (i.e., *Cx3cr1*^CreER^:iDTR system relying on tamoxifen and diptheria toxin administration), whereas we utilize CSF1R inhibitors to evaluate as a proof-of-principle whether replacement of microglia is a clinically feasible approach [55, 56]. To explore the long-term consequences of this engraftment, animals remained on control chow for an additional 6 months prior to sacrifice (~ 10 months after irradiation). At this time point, peripheral percent chimerism was evaluated via BM-collected hematopoietic stem cell (HSC) GFP expression and averaged ~ 97% in both irradiated groups (Fig. 1H).

Immunohistochemical examination of the brains ~ 10 months after irradiation and ~ 7 months after PLX3397 treatment revealed the extent of BM-derived cell parenchymal engraftment. As expected, no GFP+ cells were found in the control brains (Fig. 1D). Irradiation alone, without microglial depletion/repopulation, caused partial engraftment of GFP+ BM-derived cells, particularly in meningeal and perivascular spaces, in line with prior reports (Fig. 1D; [57, 58]). Approximately 20% of parenchymal IBA1+ cells were GFP+, indicating that irradiation alone allows for limited access and engraftment of monocytes into the CNS (Fig. 1E). On the other hand, irradiated mice treated with CSF1Ri, allowing for myeloid cell repopulation (GFP-BM REPOP mice), displayed extensive (~ 80%) GFP+ myeloid cell chimerism, essentially replacing the entire microglial tissue with BM-derived cells (Fig. 1E–F). No differences in GFP+ myeloid cell repopulation were apparent between brain regions (Fig. 1D).

### Lasting effects of irradiation on BBB integrity, neurogenesis, and cell proliferation

**Fig. 2:**
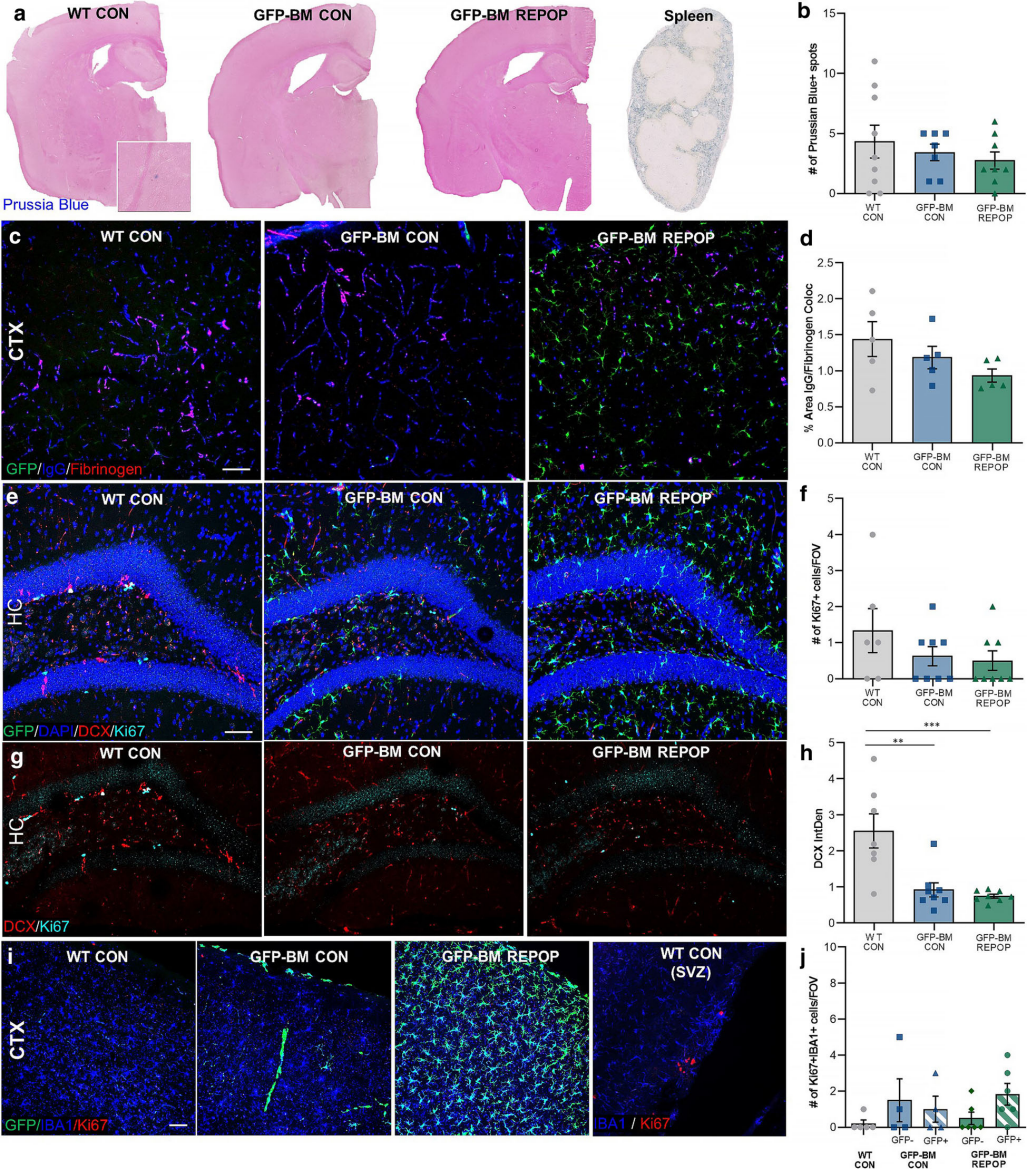
Lasting effects of irradiation on BBB integrity, neurogenesis, and cell proliferation. **a** Representative whole brain images of blood-brain barrier (BBB) integrity via Prussian blue (blue) in control (WT CON), irradiated (GFP-BM CON), and monocyte-engrafted (GFP-BM REPOP) mice. Spleen provides as a positive control. Insert shows higher-resolution images of a Prussian blue^+^ spot. **b** Quantification of number of Prussian Blue^+^ spots in the whole brain. **c** Representative immunofluorescence images of immunoglobulin (IgG, blue), fibrinogen (red), and GFP (BM-derived cells, green) in the cortex. **d** Quantification of % area of IgG and fibrinogen colocalization in the cortex. **e** Representative immunofluorescence images of neurogenesis marker doublecortin^+^ (DCX, red), proliferation marker Ki67^+^ (light blue), DAPI^+^ (blue), and GFP^+^ (green) staining. **f** Quantification of Ki67^+^ cells per FOV in the dentate gyrus of the hippocampus. **g** Representative immunofluorescence images of neurogenesis marker doublecortin^+^ (DCX, red) and proliferation marker Ki67^+^ (light blue) staining. **h** Quantification of DCX staining intensity in the dentate gyrus of the hippocampus. **i** Representative immunofluorescence images of proliferating myeloid cells stained for IBA1 (blue), proliferation marker Ki67 (red), and GFP (green). **j** Quantification of Ki67^+^ IBA1^+^ (including GFP^+^ and GFP^−^) cells per FOV. Data are represented as mean ± SEM (*n* = 5–8). **p < 0.01, ***p < 0.001. CTX cortex, HC hippocampus, SVZ subventricular zone, GFP green fluorescent protein, IBA1 ionized calcium adapter molecule 1. Scale bar ~ 50 μm

Prussian blue staining, which labels ferric iron, was performed to evaluate for the presence of microbleeds or microhemorrhages in the brain. No significant changes in Prussian blue were observed (Fig. 2A–B). Previous studies have shown that irradiation, with accompanying monocyte infiltration, has lasting effects on BBB integrity [52, 59–61]. Despite an observed influx of peripheral-derived myeloid cells, BBB integrity was not compromised, as assessed by IgG, and fibrinogen staining ~ 10 months post-irradiation (Fig. 2C–D).

Another potent and long-term effect of irradiation involves impairing neurogenesis and cell proliferation [62, 63]. In line with these studies, we observe a significant decrease in doublecortin, a marker for neuronal precursor cells/neurogenesis, in the subgranular zone of the dentate gyrus in the hippocampus, a major site of neurogenesis in the mouse brain, following irradiation, which remains reduced following monocyte infiltration (Fig. 2E–H). Monocytes are short-lived cells, with a half-life of approximately 1–7 days in humans, whereas studies have shown that microglia exhibit much slower turnover rates with an average lifespan of 4.2 years in humans [64, 65]. To assess how the engrafted monocyte population is maintained over time, we explored cell proliferation using Ki67 and found no significant differences in IBA1+ cell proliferation. In fact, we detected no to little Ki67+IBA1+ cells in the cortex across all groups, including Ki67+GFP+ cells (Fig. 2I–J). In addition to examining these known long-term effects of irradiation, these data also suggest that once peripheral donor cells engraft the CNS, they are long-lived in the brain with very little cell turnover.

### Myeloid cell characterization after long-term peripheral myeloid cell engraftment

**Fig. 3:**
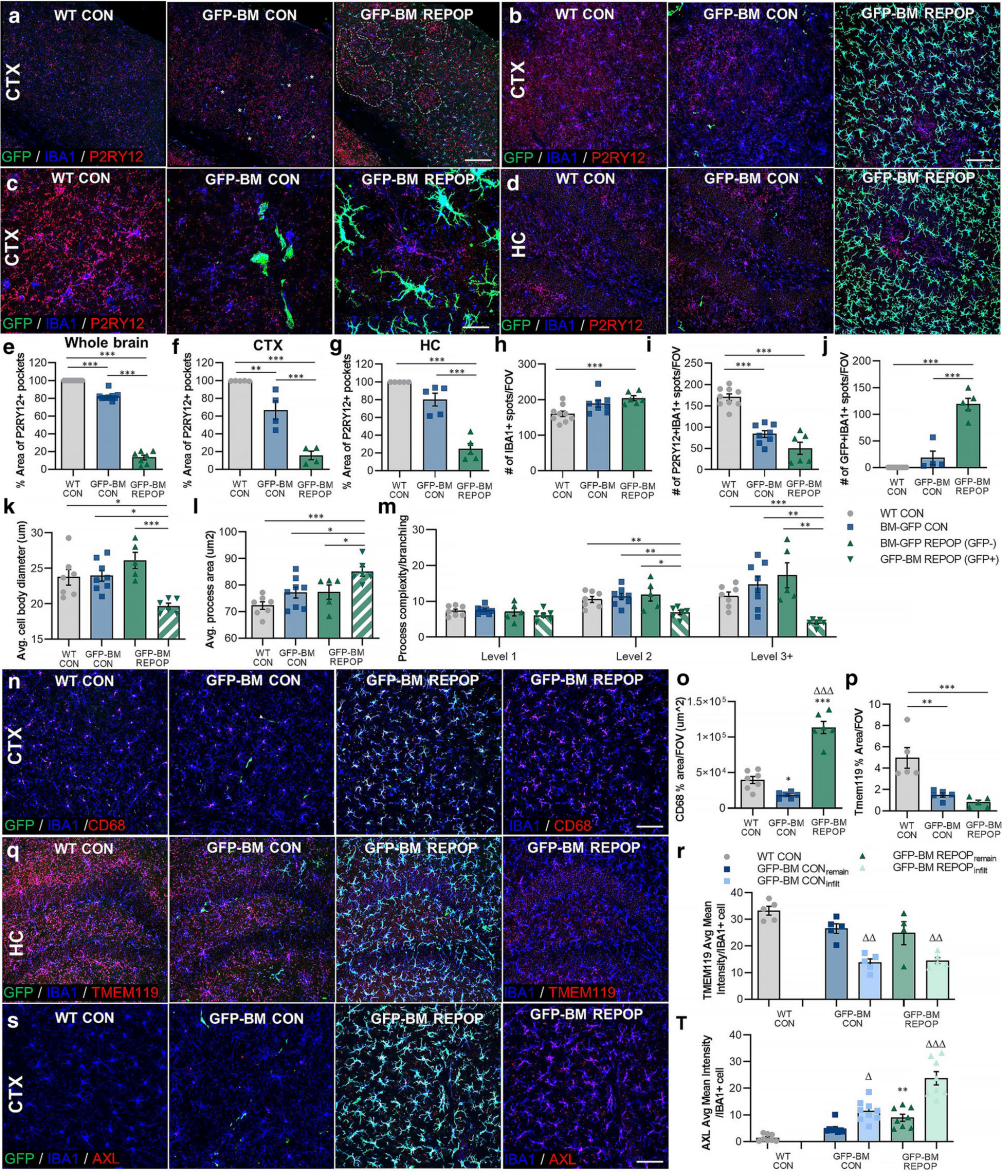
Myeloid cell characterization after long-term peripheral myeloid cell engraftment. **a**–**d** Representative immunofluorescence images of myeloid cells in the brains of control (WT CON), irradiated (GFP-BM CON), and monocyte-engrafted (GFP-BM REPOP) mice stained for common myeloid marker ionized calcium-binding adaptor molecule 1 (IBA1, blue), microglial specific marker P2RY12 (red), and GFP (BM-derived cells, green) in the cortex (**a**–**c**) and hippocampus (**d**). Higher-resolution image showing P2RY12 immunoreactivity of IBA1^+^ and GFP^+^ cells (**c**). White dotted areas indicate pockets of high P2RY12 immunoreactivity, which coincide with a lack GFP^+^ cells. **e**–**m** Quantification of P2RY12, IBA1, and GFP^+^ staining: % area of P2RY12^+^ pockets, defined also as areas lacking GFP^+^ cell infiltrates (**e**–**g**), IBA1^+^ cell number per field of view (FOV) (**h**), P2RY12^+^IBA1^+^ cell number per field of view (FOV) (**i**), GFP^+^IBA1^+^ cell number per field of view (FOV) (**j**). For these measurements, images were captured and quantified in the hippocampus. **k**–**m** Quantification of IBA1^+^ cell morphology: average cell body diameter size (**k**), average process area (**l**), and process branching complexity (**m**). For these measurements, images were captured and quantified in the hippocampus. GFP^+^ staining was also used to distinguish between remaining microglia (IBA1^+^GFP^−^) and infiltrating monocyte (IBA1^+^GFP^+^) morphologies in GFP-BM REPOP mice. **n** Representative immunofluorescence images of myeloid cells stained for IBA1 (blue), macrophage/monocyte marker CD68 (red), and GFP (green). **o** Quantification of CD68 staining area coverage per FOV. **p** Quantification of TMEM119 staining intensity in the hippocampus. **q** Representative immunofluorescence images of myeloid cells stained for IBA1 (blue), CNS myeloid cell-specific marker TMEM119 (red), and GFP (green) in the hippocampus. **r** Quantification of the average mean intensity of TMEM119 in IBA1^+^ cells per FOV, comparing remaining microglia (remain) and infiltrating BM-derived cells (infilt). **s** Representative immunofluorescence images of proliferating myeloid cells stained for IBA1 (blue), phagocytic marker AXL (red), and GFP (green). **t** Quantification of the average mean intensity of AXL in IBA1^+^ cells per FOV, comparing remaining microglia (remain) and infiltrating BM-derived cells (infilt). Data are represented as mean ± SEM (*n* = 4–9). *p < 0.05, **p < 0.01, ***p < 0.001. CTX cortex, HC hippocampus, GFP green fluorescent protein, IBA1 ionized calcium adapter molecule 1. Scale bar ~ 150 μm (**a**); ~ 75 μm (**b**, **d**, **n**, **q**); ~ 60 μm (**s**); ~ 25 μm (**c**)

Studies have shown that monocytes downregulate their canonical markers upon infiltration and differentiation in the CNS. Moreover, the loss of the homeostatic microglial signature (e.g., *Sall1*, *Pu.1*, *Tmem119*, *Cx3cr1*, and *P2ry12*) is associated with CNS disorders [66]; thus, we sought to characterize myeloid cell surface marker expression and morphology following irradiation and microglial replacement. Immunohistochemistry utilizing ionized binding adaptor molecule 1 (IBA1), a marker common to all myeloid cells, and P2RY12, a microglial specific marker, was performed on brains collected ~ 7 months following CSF1Ri-induced peripheral myeloid cell infiltration (Fig. 3A–D). Here, the remaining microglia form pockets between GFP+ spaces within the brain (Fig. 1D; Fig. 3A; outlined by white dotted lines; quantified in Fig. 3E–G). We define these pockets or islands of remaining microglia as areas of cells that are GFP− and often P2RY12+, which are only apparent in GFP-GM REPOP mice (Fig. 3A). GFP-BM REPOP mice also exhibited an elevated number of IBA1+ cells compared to controls (Fig. 3B–D and quantified in 3H). Further characterization of IBA1+ cells shows that the number of IBA1+P2RY12+ cells is decreased in GFP-BM CON and GFP-BM REPOP mice (Fig. 3I), whereas the number of IBA1+GFP+ cells are only significantly increased in GFP-BM REPOP (Fig. 3J). These data indicate that the myeloid cells that fill the brain in GFP-BM REPOP mice are BM-derived cells and do not express a classical homeostatic microglial marker. IBA1+GFP+ BM-derived myeloid cells in GFP-BM REPOP mice also displayed distinct morphological differences from IBA1+ cells in WT CON and GFP-BM CON, as well as, IBA1+GFP− cells in GFP-BM REPOP mice, in which they displayed smaller cell bodies (Fig. 3K), larger dendrites/cell processes (Fig. 3L), and reduced dendritic complexity (Fig. 3M), consistent with BM-derived cells (i.e., monocytes) exhibiting more amoeboid-like morphology compared to microglia (refer to Fig. S1A for representative morphology reconstructions).

In addition, monocyte-engrafted brains displayed significantly increased CD68, a lysosomal marker associated with a heightened activation or phagocytic state, staining compared to controls and irradiated brains (Fig. 3N–O). As expected, all IBA1+ cells in control brains exhibited extensive TMEM119 immunoreactivity (Fig. 3P, S1B), and all GFP+ cells (i.e., BM/peripheral-derived cells) displayed little to no TMEM119+ staining (Fig. 3Q). In addition, it appears that irradiation induces a significant loss of TMEM119 expression (Fig. 3P, S1B). These data indicate that the engrafting BM-derived myeloid cells, even following 7 months of residence in the brain, continue to lack microglial signature expression. Collectively, these results show that although monocytes can take up residence in the brain, they maintain a unique cell surface marker and morphological identity within the brain parenchyma, even after extended periods of residence in the brain.

After evaluating all IBA1+ cells, we next sought to delineate infiltrating monocytes from microglia unaltered by treatment, referred to as *remaining microglia*. Analysis of images in regions of extensive infiltrating monocyte engraftment or extensive remaining microglia presence shows that the loss in the microglial homeostatic signature (i.e., TMEM119) is specific to monocytes. Expression of TMEM119 in remaining microglia is not significantly altered by irradiation or repopulation (Fig. 3Q–R). In addition, we also analyzed AXL, a protein enriched in phagocytic cells, and observe significant increased expression of AXL in infiltrating monocytes in both irradiated groups, as well as, remaining microglia in GFP-BM REPOP. However, AXL expression appears most elevated in infiltrating monocytes in GFP-BM REPOP mice (Fig. 3S–T).

### Transcriptional changes following long-term peripheral myeloid cell engraftment

**Fig. 4:**
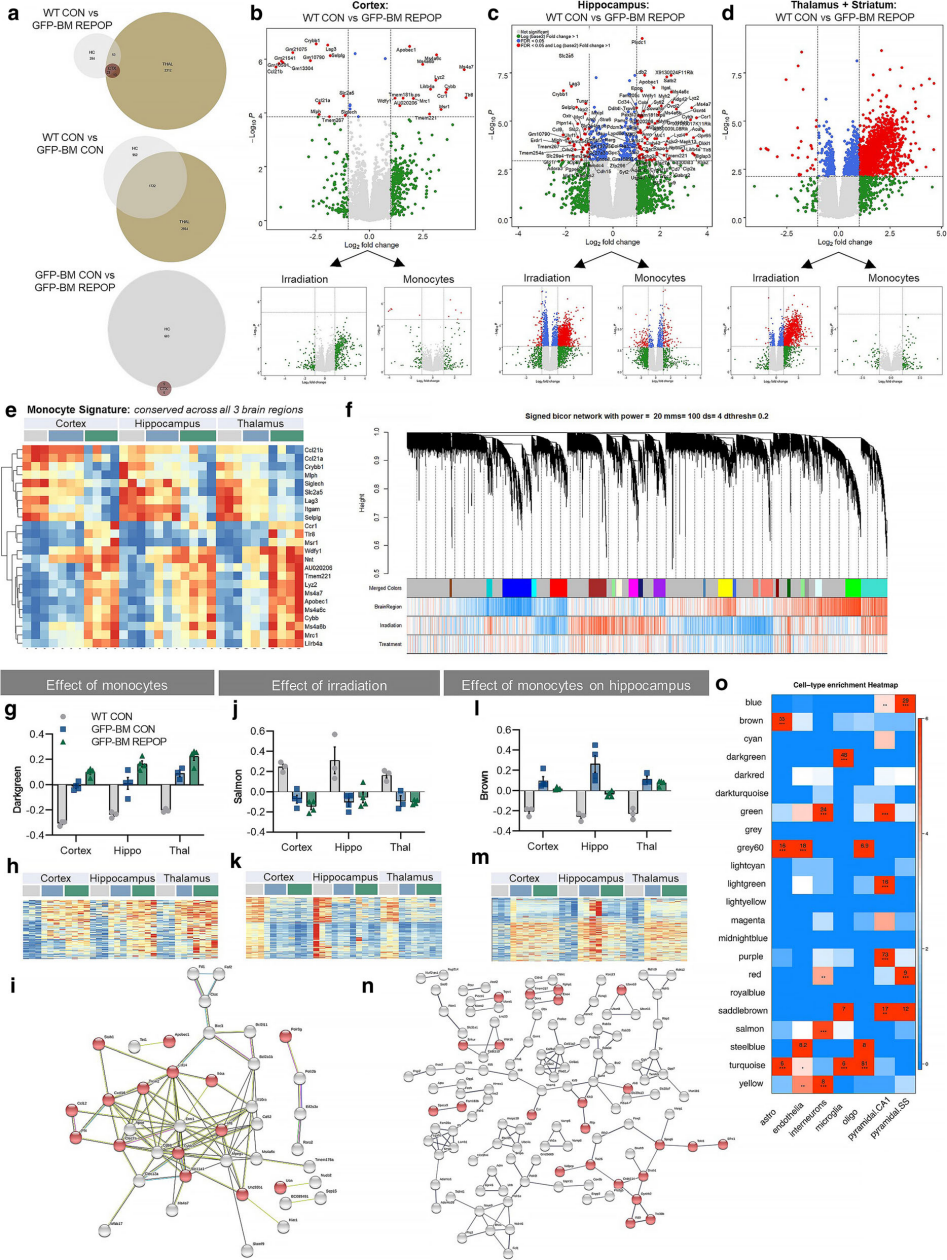
Transcriptional changes following long-term peripheral myeloid cell engraftment. **a** Venn diagrams displaying the number of differentially expressed genes (DEGs, numbers provided) generated in transcriptional comparisons between mice (control, WT CON; irradiated, GFP-BM CON; monocyte-engrafted, GFP-BM REPOP) in the hippocampus (HC, gray), cortex (CTX, red), and thalamus + striatum (THAL, tan). **b**–**d** Volcano plots displaying the fold change of genes (log2 scale) and their *p* values (−log10 scale) between WT CON vs GFP-BM REPOP in the cortex (**b**), hippocampus (**c**), and thalamus + striatum (**d**). Plots are further separated by the effect of irradiation and presence of monocytes alone. **e** Heatmap of the monocyte signature (selected DEGs conserved in all three brain regions in monocyte-engrafted mice). **f** Signed bicor network shows the effects of brain region (BrainRegion), irradiation, and treatment, which are separated into distinct colored modules. **g**–**i** Monocyte signature: Module eigengene trajectory of darkgreen (**g**), heatmap of gene expression value in darkgreen (**h**), and a STRING interaction map of darkgreen (**i**). Red nodes indicate genes enriched in the gene ontology (GO) term defense response. (**j**–**k**) Irradiation: Module eigengene trajectory of salmon (**j**) and heatmap of gene expression value in salmon (K). **l**–**n** Effects of monocytes specific to the hippocampus: Module eigengene trajectory of red (**l**), heatmap of gene expression value in red (**m**), and a STRING interaction map of red (**n**). Red nodes indicate genes enriched in the GO term cilium organization. **o** Cell-type enrichment heatmap displays genes associated with specific cell types within a given color module. Values provided indicate the number of genes within the network associated with that a specific cell type. *** = 6+ genes. ** = 3+ genes.

After detecting alterations in myeloid cell expression, we continued our investigations by addressing the impact of long-term peripheral myeloid cell engraftment at the transcriptional level. Since previous studies have shown that microglial heterogeneity is detected in a distinct brain region-specific manner by bulk tissue RNA sequencing (RNAseq) [67–70], we performed RNAseq analysis on three brain regions (cortex, hippocampus, and thalamus/striatum). Gene expression can be explored at http://rnaseq.mind.uci.edu/green/long-term_monocytes/. To initially identify gene expression changes due to both irradiation and the brain-wide presence of monocytes, we compared the transcriptomes of GFP-BM REPOP mice to controls (WT CON), which resulted in 2695 differentially expressed genes (DEGs; FDR < 0.05) across all brain regions. Notably, the effects were most prominent in the thalamus (2401 DEGs), followed by the hippocampus (370 DEGs), with few DEGs detected in the cortex (36 DEGs; Fig. 4A). We next examined transcriptional changes due to irradiation alone (i.e., WT-CON vs. GFP-BM CON), and due to the presence of monocytes (i.e., GFP-BM CON vs. GFP-BM REPOP; Fig. 4A) and display the results as volcano plots (Fig. 4B–D). Separating the effects of irradiation and monocyte infiltration, we observe that the hippocampus and thalamus are more vulnerable to irradiation-induced gene changes, compared to the cortex, which shows little to no changes. Of note, the most significant transcriptional changes due to monocyte infiltration occur exclusively in the hippocampus. Overall, these results demonstrate that different brain regions exhibit selective vulnerabilities to irradiation and the infiltration of monocytes. Using the DEGs present in the transcriptional comparison between WT CON vs. GFP-BM REPOP mice across all three brain regions, we were able to build a unique long-term monocyte-related signature (Fig. 4E). Among these genes most prominently upregulated in monocyte-engrafted mice, we identified *Apobec1*, the chemokine receptor *Ccr1*, the C-type lectin *Mrc1*, and several members of the Ms4a cluster (*Ms4a6b*, *Ms4a6c*, and *Ms4a7*). In this comparison, known microglial specific genes were also downregulated, including *Crybb1*, *Slc2a5*, *Siglech* (selected genes shown in Fig. S2A).

To understand and place transcriptional changes associated with microglial-monocyte replacement and irradiation within three distinct brain regions on a broader system-level scale, we next conducted weighted gene co-expression network analysis (WGCNA) [49]. Twenty-two distinct modules were identified and given color-based names (Fig. 4F). The correlation between modules is shown in Fig. S2B, highlighting modules that behave similarly to one another. We initially focused on modules that exhibited the highest correlation with monocyte presence in the brain (“treatment” variable) and identified a single module: darkgreen (Fig. 4G–I). Eigengene values for darkgreen showed consistent increases in GFP-BM REPOP mice across all three brain regions, reflected in the provided heatmap (Fig. 4H). Furthermore, cell-type enrichment analysis (Fig. 4O) demonstrated that darkgreen genes are myeloid-enriched and included previously identified peripheral myeloid-related genes: *Apobec1*, *Apoe*, *Clec7a*, *Ms4a6c*, and *Ms4a7*. Accordingly, pathway analyses listed the top Biological Process Gene-Ontology (GO) terms: *response to other organism*, *defense response to protozoan*, *response to external stimulus*, *defense response*, and *positive regulation of cytokine production*. Genes in the darkgreen module are displayed in a functional protein-protein interaction network highlighting genes (in red) in *defense response* (Fig. 3I). In addition to identifying a monocyte-specific signature module, we also identified several modules associated with irradiation across all three brain regions. Here, we provide one module: salmon (Fig. 4J–K). Cell-type enrichment analysis shows that the salmon module is significantly enriched with interneuron genes (Fig. 4O). Functional annotation of the salmon module shows that it is enriched in the following top GO terms: *cellular process* and *gene expression*, and Reactome Pathways: *Metabolism of RNA*, *Processing of Capped intron-Containing Pre-mRNA*, *mRNA Splicing*, and *RNA Polymerase II Transcription*. These findings are consistent with the known effects of irradiation, including DNA damage and repair [71], and recent studies have shown that splicing factors are recruited to sites of DNA damage [72]. Several modules indicate that monocyte engraftment leads to a partial recovery of irradiation-induced effects, specifically in the hippocampus. Here, we show one module: brown (Fig. 4L–N). Cell enrichment analysis revealed the brown module to be highly enriched for astrocyte-expressed (astro) genes (Fig. 4O). The top GO terms identified in this module were *cilium organization*, *plasma membrane bounded cell projection assembly*, *cilium assembly*, and *cell projection organization*. Genes in the brown module are displayed in a functional protein-protein interaction network highlighting genes (in red) in *cilium organization* (Fig. 4N). Cilia are small microtubule-based signaling projections expressed on virtually all cells, including neurons and glia. Cilium function is implicated in sensing the extracellular environment, signal transduction, and regulating cell division/cell cycle [73]. Recent studies also indicate that cilia and cilia-associated proteins may play a role in neuronal development and DNA damage and repair [74]. Together, these data indicate that irradiation and the presence of repopulating monocytes play a role in altering astrocyte and neuronal-related genes in the brain, as well as, exhibit distinct effects in different brain regions, including the cortex and hippocampus.

### The differential and brain region-dependent effects of long-term peripheral myeloid cell engraftment on astrocytic and neuronal properties

**Fig. 5:**
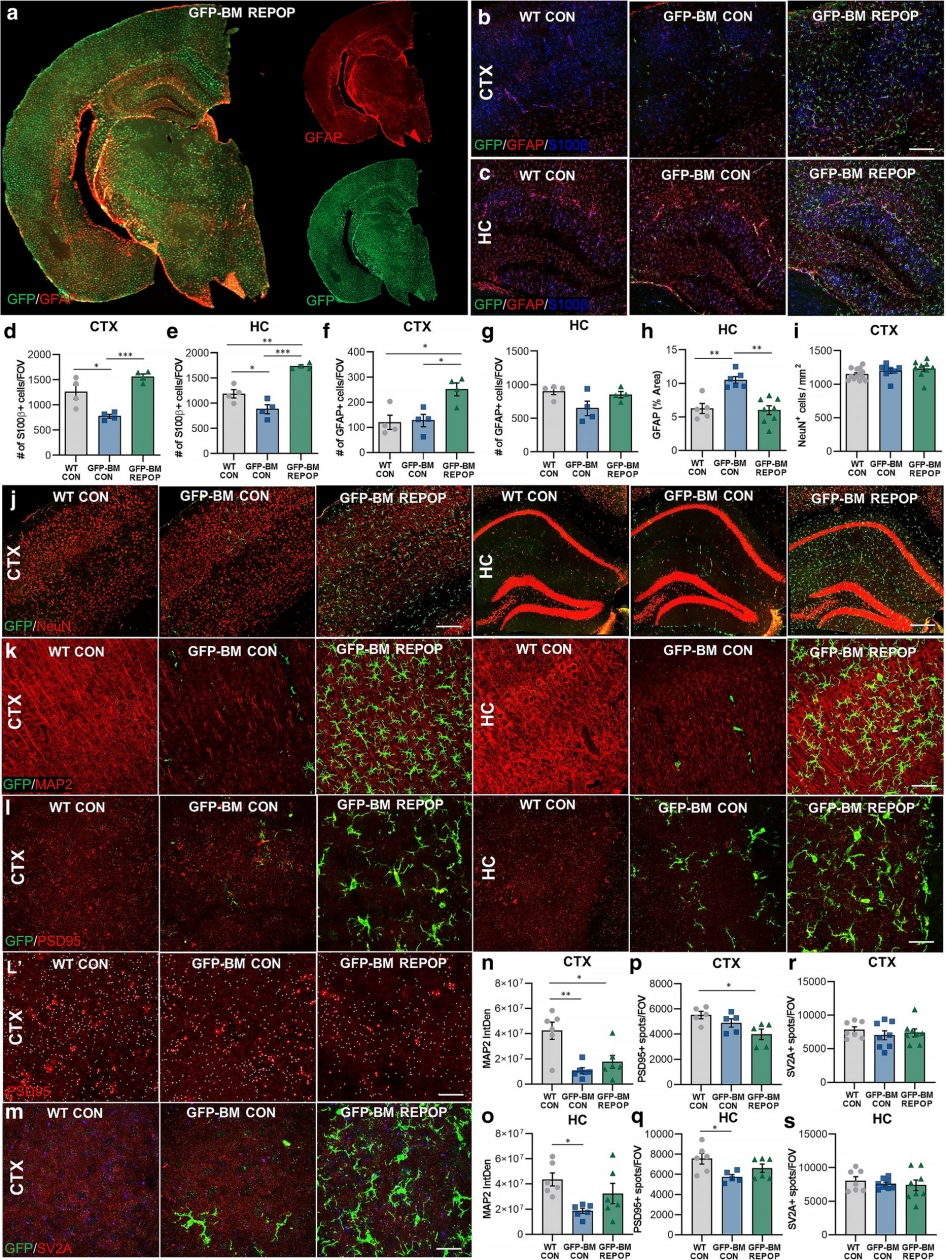
The differential and brain region-dependent effects of long-term peripheral myeloid cell engraftment on astrocytic and neuronal properties. **a** Representative whole brain images of GFP (green) and common astrocyte marker glial fibrillary acidic protein (GFAP, red) cell deposition in monocyte-engrafted mice. **b**–**c** Representative immunofluorescence images of different astrocyte subtypes stained for GFAP (red) and S100β (blue) in the in the cortex (B) and hippocampus (C) of control (WT CON), irradiated (GFP-BM CON), and monocyte-engrafted (GFP-BM REPOP) mice. GFP (green) stains BM-derived cells. **d**–**g** Quantification of S100β^+^ and GFAP^+^ cell number in the cortex (D, F) and cell number in the hippocampus (**e**, **g**). **h** Quantification of GFAP staining intensity in the hippocampus. (**i**) Quantification of NeuN^+^ cell number in the whole cortex. (**j**) Representative immunofluorescence images stained for neuronal marker NeuN (red) and GFP (green, BM-derived cells) in the cortex and hippocampus. (**k**) Representative immunofluorescence images of microtubule associated protein 2 (MAP2, red) and GFP (green, BM-derived cells) in the cortex and hippocampus. (**l**) Representative immunofluorescence brain images of synaptic marker PSD95 (postsynaptic density protein 95, red) and GFP (green, BM-derived cells) in the cortex and hippocampus. L’ provides representative images of spots modeling using Imaris spots module software overlaid on PSD95^+^ staining. (**m**) Representative immunofluorescence brain images of synaptic marker SV2A (synaptic vesicle glycoprotein 2A, red) and GFP (green, BM-derived cells) in the cortex. (**n**–**s**) Quantification of the integrated density of MAP2 staining in the cortex (**n**) and hippocampus (**o)**, total number of PSD95^+^ spots per field of view in the cortex (**p**) and hippocampus (**q**), and total number of SV2A^+^ spots per field of view in the cortex (**r**) and hippocampus (**s**). Data are represented as mean ± SEM (*n* = 4–9). *p < 0.05, **p < 0.01. ***p < 0.001. CTX cortex, HC hippocampus, GFP green fluorescent protein, IBA1 ionized calcium adapter molecule 1. Scale bar ~ 150 μm (B–C, **j**–left panel); ~ 200 μm (**j**–right panel); ~40 μm (**k**); ~ 30 μm (**l**, **m**) ~ 15 μm (L’)

To further explore these differential effects, we next assessed astrocytes and neurons in various regions of the brain using immunohistochemical analysis. Astrocytes, the other major glial cell of the CNS, were stained using astrocyte markers glial fibrillary acidic protein (GFAP) and S100β (Fig. 5A–C; Fig. S3A, B). S100β+ (Fig. 5D; Fig. S3A) and GFAP+ (Fig. 5F; Fig. S3B) cells were significantly elevated in the cortex of GFP-BM REPOP mice. A significant elevation in S100β+ cells was also found in hippocampus in these mice (Fig. 5E), but no differences were detected in the number of GFAP+ cells (Fig. 5G), indicating that monocyte-engrafted brains show heightened astrocyte numbers for specific astrocyte subsets in a brain region-dependent manner. Furthermore, we observed a significant increase in GFAP+ staining intensity in the hippocampus of BM-GFP CON mice (Fig. 5H), indicating that astrocytes in the hippocampus of irradiated mice are not elevated in cell number, but appear more activated, and that this activation is reversed by monocyte engraftment. These data are in line with transcriptional findings that monocyte engraftment reverses irradiation-induced astrocyte-associated gene changes (Fig. 4L–N).

In addition to astrocyte alterations, we also explored neurons and associated structural properties (e.g., axons and synapses). No gross differences were detected in neuronal numbers following irradiation or long-term monocyte engraftment, as assessed by neuronal marker NeuN (Fig. 5I–J). However, as has been previously reported by others, microtubule-associated protein 2 (MAP2) was significantly decreased in both brain regions (i.e., cortex, hippocampus) in GFP-BM CON mice (Fig. 5K, N–O) [75–77]. Consistent with previous findings in the hippocampus, a significant reduction in MAP2 expression was not seen in GFP-BM REPOP mice, indicating a reversal in irradiation-induced changes following monocyte engraftment. Next, we explored synaptic alterations using postsynaptic density 95 (PSD-95; Fig. 5L–L’), a major scaffolding protein expressed on excitatory synapses, and synaptic vesicle glycoprotein 2A (SV2A; Fig. 5M), a synaptic vesicle protein expressed ubiquitously in the CNS present on GABAergic and glutamatergic presynaptic terminals [78]. A significant loss of PSD95+ synapses was found in GFP-BM REPOP mice in the cortex, and a significant loss was found in GFP-BM CON mice in the hippocampus but not in GFP-BM REPOP mice (Fig. 5L–L’, P–Q). No significant alterations in SV2A staining were detected (Fig. 5M, R–S). Together, these data indicate that irradiation and monocyte engraftment can have differential effects on astrocytic and neuronal properties in a brain region-dependent manner. These data also confirm that in the hippocampus, monocyte engraftment appears to reverse some irradiation-induced effects.

### Effects of long-term peripheral myeloid cell engraftment on behavior and cognition

**Fig. 6:**
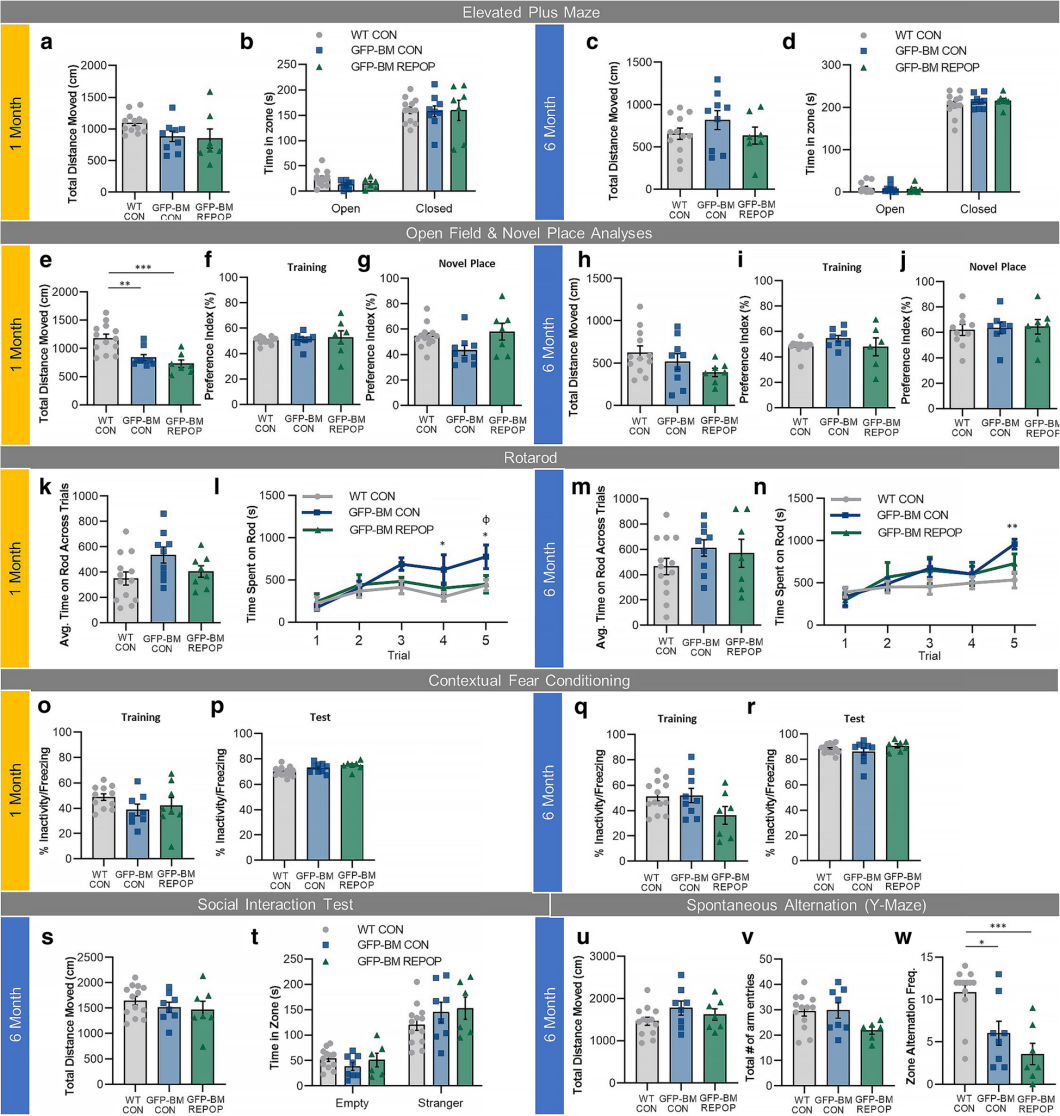
Effects of long-term peripheral myeloid cell engraftment on behavior and cognition. (**a**–**d**) Quantification of elevated plus maze performance at 1 month (**a**–**b**) and 6 months (**c**–**d**) post CSF1Ri treatment in control (WT CON), irradiated (GFP-BM CON), and monocyte-engrafted (GFP-BM REPOP) mice as assessed by time spent in open and closed arms (**b**, **d**). Total distance moved was also measured to control for locomotive changes (**a**, **c**). (**e**–**j**) Quantification of open field (**e**, **h**) and novel place recognition (**g**, **j**) performance at 1 month (**e**–**g**) and 6 months (**h**–**j**) as assessed by total distance moved (**e**, **h**) and percent preference index (**f**–**g**, **i**–**j**), respectively. Preference index is the ratio of the amount of time spent exploring any one of two objects (*A* or *B*) in the training phase or the novel-placed object (*C*) in the test phase over the total time spent exploring both objects, i.e. *C*/(*B* + *C*) × 100 in the test phase. Quantification of training also provided (**f**, **i**). (**k**–**n**) Quantification of rotarod performance at 1 months (**k**–**l**) and 6 months (**m**–**n**). (**o**–**r**) Quantification of contextual fear conditioning at 1 months (**o**–**p**) and 6 months (**q**–**r**) post CSF1Ri-induced monocyte engraftment as assessed by percentage of inactivity at testing (**p**, **r**). Quantification of training also provided (**o**, **q**). **s**–**t** Quantification of social interaction test performance at 6 months post CSF1Ri treatment as assessed by time spent in an empty zone or zone containing a stranger mouse (**t**). Total distance moved was also measured to control for locomotive changes (**s**). **u**–**w** Quantification of spontaneous alternation (Y-maze) performance at 6 months post CSF1Ri treatment as assessed by zone alternation frequency (**w**). Total distance moved and total number of arm entries was also measured to control for locomotive changes (**u**) and motivation/movement (**v**), respectively. *p < 0.05. ***p < 0.001

After observing changes at the cellular level, we next sought to determine whether irradiation and/or myeloid cell replacement leads to short- or long-term alterations in behavior or cognition. To accomplish this, mice underwent behavioral and cognitive testing 1 and 6 months following CSF1Ri-induced repopulation (Fig. 1A). Mice were first tested on the elevated plus maze (Fig. 6A–D). Neither irradiated (GFP-BM CON) nor repopulated (GFP-BM REPOP) mice exhibited changes in anxiety during this task at 1 month (Fig. 6B) or 6 months (Fig. 6D) post-repopulation compared to controls (WT CON), and no motor differences were recorded during this task (Fig. 6A, C). Despite the lack of changes in the elevated plus maze, we did observe significant differences during open-field analyses in distance traveled (Fig. 6E) in both transplanted groups compared to WT CON at 1 month, showing that irradiation impacts locomotion. However, these significant alterations were transient and not detected at 6 months (Fig. 6H). Evaluations of locomotive activity utilizing a rotarod apparatus show that irradiation does slightly impact average motor function over all trials (*p* = 0.055) at 1 month (Fig. 6K). GFP-BM CON mice improve over time and stay on longer compared to controls (Fig. 6L); however again, these alterations were transient and not detected at 6 months (Fig. 6M–N). To assess recognition memory, mice were tested using the novel place recognition test. In this task, we observed no significant differences between groups (Fig. 6F–G, I–J); however, we do observe a slight increase in memory as measured by preference index in GFP-BM REPOP mice at 1 month. To further investigate these memory alterations, hippocampal-dependent learning and memory was assessed using the contextual fear conditioning apparatus (Fig. 6O–R). No significant differences were detected between groups as measured by % freezing at 1 month (Fig. 6O–P) and 6 months (Fig. 6Q–R), indicating that hippocampal learning and memory is not altered by irradiation or the presence of monocytes. Additional behavior and cognitive testing were added at 6 months, including Crawley’s sociability test (Fig. 6S–T) and spontaneous alternation Y-maze (Fig. 6U–W). No significant differences in general sociability were found between groups, as assessed by time spent in empty and stranger mouse-filled chambers (Fig. 6T). The spontaneous alternation Y-maze serves as an index for active retrograde working memory, which relies on the proper functioning of many brain regions, including the hippocampus and cortex [79]. Here, we observed the most overt behavioral alterations as a result of irradiation. Both GFP-BM CON and GFP-BM REPOP exhibited significantly reduced zone alternation frequency compared to WT CON (Fig. 6W), indicating that working memory may be impaired by irradiation. It should be noted that the number of total arm entries was slightly reduced in GFP-BM REPOP mice (Fig. 6V). Together, these findings indicate that by most behavioral tests, irradiation and monocyte engraftment result in little changes to behavior and cognition; however, there appears to be a significant long-term deficit in retrograde working memory, which is associated with the temporal lobe and prefrontal cortex.

## Discussion

Monocytes derive from myeloid precursor cells in the BM and possess the ability to differentiate into tissue-specific macrophages in various organs and tissues, including the CNS. However, the role of these blood-derived myeloid cells in the CNS along with their ability to fill the microglial niche and long-term effects on the brain remain unclear. Here, we developed a paradigm using whole body irradiation, BM transplant, and pharmacological inhibition of CSF1R to achieve near complete and long-term engraftment of peripheral derived myeloid cells (i.e., monocytes) in the brain. Previous studies have shown that the microglial population relies on CSF1R signaling for survival and, thus, can be eliminated and repopulated using CSF1Ri administration and withdrawal, respectively [7, 16, 18, 21, 53, 80]. Here, our data show that CSF1R inhibition following whole body irradiation results in the near complete (~ 80%) replacement of microglia with monocytes. These data indicate that monocytes are capable of outcompeting endogenous CSF1Ri-resistant microglia under these conditions. Previous data has shown that microglia survive cranial irradiation, however do exhibit increased activation and decreased cell number [81], and that irradiation induces loss of proliferating cells, including progenitor/stem and microglial cells [60]. Thus, in order to induce complete monocyte engraftment, it appears that the brain must possess an empty microglial niche or microglial cells that lack the ability to proliferate/self-renew. In line with this, Bruttger et al. showed that genetic microglial ablation of BM chimeric mice (i.e., exposed to irradiation) resulted in repopulation of the microglial pool with BM-derived myeloid cells [54]. Furthermore, recent findings have demonstrated that chronic microglial depletion, using a genetic model of partial microglia deficiency, or CSF1Ri treatment (during irradiation) leads to monocyte engraftment in the CNS [22, 82].

Recent data demonstrate that monocytes are capable of filling the microglial niche [83] and taking up residence in the brain; however, these peripheral-derived cells remain phenotypically, transcriptionally, epigenetically, and functionally distinct from their microglial counterparts [22, 24, 82, 84, 85]. In this study, we observe that engrafted monocytes maintain a loss of homeostatic microglial signature markers and an increase in markers associated with phagocytosis. To address whether these differences translate into long-term consequences in the brain, we evaluated mice for transcriptional, cellular, and behavioral changes 6 months following monocyte engraftment into the CNS.

Transcriptional analysis reveals that the thalamus and hippocampus are the most vulnerable brain regions to irradiation. Further stratification of these comparisons teasing apart irradiation and monocyte-specific effects shows that the hippocampus is the only brain region that exhibits significant translational changes in response to monocyte infiltration. Notably, WGCNA analysis identified several modules elucidating a reversal in irradiation-induced effects following monocyte engraftment, occurring exclusively in the hippocampus. Pathway analysis of these modules reveals mechanisms involving cilium organization and assembly. A recent study using granulocyte colony-stimulating factor (G-CSF) receptor knockout mice and BM transplant show that BM cells (responding to G-CSF) home to the irradiated brain and promote brain repair and neural progenitor cell proliferation [86]. Recent reports have demonstrated that brain-engrafted monocytes exhibit a distinct gene signature compared to microglia [22, 82]. Here, we highlight a distinct gene signature of engrafted monocytes compared to microglia following 6 months of recovery from CSF1Ri administration and 10 months following irradiation. Our monocyte signature includes an upregulation in *Ccr1*, *Ms4a6b*, *Ms4a6c*, *Ms4a7*, *AU020206*, *Apobec1*, *Lyz2*, *Mrc1*, *Tmem221*, *Tlr8*, *Lilrb4a*, *Msr1*, *Nnt*, and *Wdfy1*, coinciding with a downregulation of *Siglech*, *Itgam*, *Selpig*, *Lag3*, *Slc2a5*, *Mlph*, *Crybb1*, *Ccl21a*, and *Ccl21b*. Notably, this differential gene signature was not detected in brain-extracted monocytes [22], except for an overlap in expression of *Tlr8* between the two datasets, showing how extraction techniques and/or time in the brain play critical roles in monocyte gene expression (i.e., 6 weeks vs 6 months). *Tlr8* is a member of the toll-like receptor family and plays an important role in pathogen recognition and innate immunity. It is also a gene that is predominantly expressed on peripheral blood leukocytes.

On the cellular level, brain region-specific differences in irradiation and monocyte engraftment were apparent. Astrocyte numbers were reduced by irradiation, but then elevated (past control levels) in monocyte-engrafted mice in both the cortex and hippocampus. These findings highlight not only irradiation-induced deficits in astrocytes, but also compensatory responses for this loss following CSF1Ri-induced monocyte engraftment. Together, these data provide evidence of monocyte-astrocyte crosstalk; it could be possible that monocytes are involved in regulating astrocytes or that astrocytes respond to monocyte engraftment in the brain. In line with this, a previous study highlights cross-regulation between astrocyte proliferation and monocyte invasion during scar formation after brain injury. However, contrary to our data, they show that reducing monocyte invasion using CCR2^−/−^ mice results in elevated reactive astrocyte proliferation [87]. In addition to astrocyte changes, alterations in MAP2 were also found. MAP2 was significantly reduced by irradiation in the cortex and hippocampus and returned to control levels in the hippocampus of monocyte-engrafted mice, similar to previous transcriptional patterns. MAP2 is important for microtubule stabilization, neural plasticity, and normal neuronal cytoskeletal structure; thus, this data could reflect alterations in dendritic spine structure or neurite growth. In agreement, a previous study has shown that cranial irradiation results in significant decreases in dendritic spine density in pyramidal neurons in the hippocampus [88]. Thus, dendritic spine structure or neurite growth may be lost during irradiation and returned to control levels at the facilitation of monocytes in the hippocampus. However, MAP2 also serves as a ciliary marker. In the adult brain, neurons and astrocytes contain one primary cilium, a small microtubule-based signaling process, considered the cellular “antennae” for detecting extracellular signals. Studies have shown that primary cilia are responsible for regulating cell division, development, and tissue regeneration (including DNA damage and repair) [73, 74]. Further work is needed to explore the effects of irradiation and monocyte engraftment on primary cilia, but these alterations in MAP2 staining could also suggest that replacing microglia with monocytes may help facilitate primary cilia integrity lost due to irradiation. Evaluation of synaptic markers reveals brain region-specific vulnerability to irradiation and monocyte engraftment. In the hippocampus, we observe that irradiation can lead to synaptic deficits, as seen by a loss in PSD95^+^ puncta, even ~ 10 months after irradiation, which is slightly improved by monocyte engraftment. However, in the cortex, we only observe a significant loss of PSD95^+^ puncta in monocyte-engrafted mice, indicating that monocytes may phagocytose these synaptic elements. In contrast, a recent report showed that monocytes exhibit a reduced capacity to phagocytose synaptic compartments, although it should be noted that these investigators used a different treatment paradigm (mice were treated for 21 days with CSF1Ri during three rounds of irradiation) and evaluated 4 weeks after irradiation, which could explain the discrepancy. No differences in synaptic marker SV2A were found, indicating that the synaptic alterations observed in monocyte-engrafted mice could be specific to postsynaptic terminals. Since microglia are involved in remodeling synapses, specifically in pruning presynaptic inputs [89], further work is needed to explore and elucidate these long-term engrafted monocyte-synapse interactions.

By behavioral and cognitive task evaluation, we observe few significant differences in behavior in control compared to monocyte-engrafted mice, consistent with previous findings by Cronk et al. that the presence of 48% CNS engraftment of monocytes does not significantly alter cognitive function [22]. The most prominent changes in behavior were found as a result of irradiation, which lead to alterations in locomotive activity as measured by total distance traveled at 1 month and reduced working memory as measured by a spontaneous alternation task, in both irradiated and monocyte-engrafted mice. In line with these findings, several studies have shown that cranial irradiation disrupts behavioral and cognitive function in rodents, including motor activity and hippocampal and non-hippocampal-dependent cognitive tasks [90, 91]. Interestingly, we do observe a decrease in cortical MAP2 and PSD95 staining, which could indicate brain region-specific vulnerability to irradiation, specifically the cortex. In agreement with this, a recent study has shown that in humans cerebral cortical regions are selectively vulnerable to radiation-related atrophy [92] and a study in rats showed that radiation induces deficits in cortical synaptic plasticity [93]. Since the spontaneous alternation task relies on various areas of the cortex, as well as the hippocampus, it could be possible that irradiation results in deficits involving the functional integration of these two brain regions. In line with this data, a recent study has shown that following sub-lethal irradiation brain injury, patients experience longitudinal changes in the functional connectivity of hippocampal-related cortices [94]. Our study indicates that in the hippocampus, monocyte engraftment can help resolve irradiation-induced deficits, however, in the cortex, may further exacerbate these deficits.

## Conclusions

In this study, we provide evidence that irradiation and monocyte engraftment elicit brain region-dependent alterations and show that irradiation-induced changes can be reversed by the replacement of microglia with monocytes in the hippocampus. Most importantly, we highlight the long-term impact of monocyte engraftment on the CNS. Our data provide evidence that near complete and long-term peripheral myeloid cell engraftment can be achieved, providing a model to deliver therapeutically relevant cells and molecules to the brain; however, the next steps involving therapeutic use should be aware of the differential and sometimes detrimental effects of monocyte engraftment in the CNS. We propose that in developing improved methods of cell delivery to the brain, investigators should focus on methods that do not involve whole-body irradiation. These methods could include promising developments in irradiation technology such as flash irradiation, which are less harmful to the CNS environment. Successful replacement of the microglial tissue with BM-derived cells could have broad implications for many neurological diseases, including those associated with myeloid cell dysfunction, such as Alzheimer’s disease and multiple sclerosis, as well as, provide a route for brain-wide therapeutic gene delivery.

## Supplementary information

**Additional file 1: Figure S1.** Related to Figure 3: Myeloid cell characterization after long-term peripheral myeloid cell engraftment. (A) Representative immunofluorescence and high resolution images of microglial morphology modeling (utilizing the Imaris Filaments module) of myeloid cells in the brains of control (WT CON), irradiated (GFP-BM CON), and monocyte-engrafted (GFP-BM REPOP) mice stained for common myeloid marker ionized calcium binding adaptor molecule 1 (IBA1, blue) and GFP (BM-derived cells, green) in the cortex. (B) Representative immunofluorescence single stain image of TMEM119 (red), a unique marker for microglia. Scale bar: ~100 μm (A, 20x); ~15 μm (A, 63x); ~75 (B). **Figure S2.** Related to Figure 4: Transcriptional changes following long-term peripheral myeloid cell engraftment. (A) Quantification of RPKM values for the top upregulated and downregulated genes in monocyte-engrafted mice across all three brain regions. All RPKM values can be explored at http://rnaseq.mind.uci.edu/green/long-term_monocytes/ (B) Eigengene network displaying the correlation between color modules (n = 32). Data are represented as mean ± SEM (n=4). **Figure S3.** Related to Figure 5: The differential and brain region-dependent effects of long-term peripheral myeloid cell engraftment on astrocytic and neuronal properties. (A-B) Representative immunofluorescence single stain images of different astrocyte subtypes stained for S100β (red, A) and GFAP (red, B) in the hippocampus of control (WT CON), irradiated (GFP-BM CON), and monocyte-engrafted (GFP-BM REPOP) mice. Scale bar: ~150 μm (A); ~75 μm (B,D,N,Q); ~60 (S); ~25 μm (C).

## Data Availability

The Fastq files and processed data matrices were deposited in GEO with the accession ID GSE157593. Gene expression datasets supporting the conclusions of this article are available at http://rnaseq.mind.uci.edu/green/long-term_monocytes/.
