# Supplementary material for: Effects of long-term and brain-wide colonization of peripheral bone marrow-derived myeloid cells in the CNS
Source: J Neuroinflammation. 2020 Sep 20;17:279. doi: 10.1186/s12974-020-01931-0 (PMC7504855; doi:10.1186/s12974-020-01931-0)
Supplement: Supplementary file 1 — Additional file 1: Figure S1. Related to Figure 3: Myeloid cell characterization after long-term peripheral myeloid cell engraftment. (A) Representative immunofluorescence and high resolution images of microglial morphology modeling (utilizing the Imaris Filaments module) of myeloid cells in the brains of control (WT CON), irradiated (GFP-BM CON), and monocyte-engrafted (GFP-BM REPOP) mice stained for common myeloid marker ionized calcium binding adaptor molecule 1 (IBA1, blue) and GFP (BM-derived cells, green) in the cortex. (B) Representative immunofluorescence single stain image of TMEM119 (red), a unique marker for microglia. Scale bar: ~100 μm (A, 20x); ~15 μm (A, 63x); ~75 (B). Figure S2. Related to Figure 4: Transcriptional changes following long-term peripheral myeloid cell engraftment. (A) Quantification of RPKM values for the top upregulated and downregulated genes in monocyte-engrafted mice across all three brain regions. All RPKM values can be explored at http://rnaseq.mind.uci.edu/green/long-term_monocytes/ (B) Eigengene network displaying the correlation between color modules (n = 32). Data are represented as mean ± SEM (n=4). Figure S3. Related to Figure 5: The differential and brain region-dependent effects of long-term peripheral myeloid cell engraftment on astrocytic and neuronal properties. (A-B) Representative immunofluorescence single stain images of different astrocyte subtypes stained for S100β (red, A) and GFAP (red, B) in the hippocampus of control (WT CON), irradiated (GFP-BM CON), and monocyte-engrafted (GFP-BM REPOP) mice. Scale bar: ~150 μm (A); ~75 μm (B,D,N,Q); ~60 (S); ~25 μm (C). [file 12974_2020_1931_MOESM1_ESM.docx]

**Supplementary Material**

**Figure S1. Related to Figure 3: Myeloid cell characterization after long-term peripheral myeloid cell engraftment.**

(A) Representative immunofluorescence and high resolution images of microglial morphology modeling (utilizing the Imaris Filaments module) of myeloid cells in the brains of control (WT CON), irradiated (GFP-BM CON), and monocyte-engrafted (GFP-BM REPOP) mice stained for common myeloid marker ionized calcium binding adaptor molecule 1 (IBA1, blue) and GFP (BM-derived cells, green) in the cortex.

(B) Representative immunofluorescence single stain image of TMEM119 (red), a unique marker for microglia, staining.

Scale bar: ~100 µm (A, 20x); ~15 µm (A, 63x); ~75 (B).

**Figure S2. Related to Figure 4: Transcriptional changes following long-term peripheral myeloid cell engraftment.**

(A) Quantification of RPKM values for the top upregulated and downregulated genes in monocyte-engrafted mice across all three brain regions.

All RPKM values can be explored at <http://rnaseq.mind.uci.edu/green/long-term_monocytes/>

(B) Eigengene network displaying the correlation between color modules (n = 32).

Data are represented as mean ± SEM (n=4).

**Figure S3. Related to Figure 5: The differential and brain region-dependent effects of long-term peripheral myeloid cell engraftment on astrocytic and neuronal properties.**

(A-B) Representative immunofluorescence single stain images of different astrocyte subtypes stained for S100β (red, A) and GFAP (red, B) in the hippocampus of control (WT CON), irradiated (GFP-BM CON), and monocyte-engrafted (GFP-BM REPOP) mice.

Scale bar: ~150 µm (A); ~75 µm (B,D,N,Q); ~60 (S); ~25 µm (C).
